# Adverse perinatal outcomes for obese women are influenced by the presence of comorbid diabetes and hypertensive disorders

**DOI:** 10.1038/s41598-019-46179-8

**Published:** 2019-07-05

**Authors:** Evelyne M. Aubry, Stephan Oelhafen, Niklaus Fankhauser, Luigi Raio, Eva L. Cignacco

**Affiliations:** 1 Health Department, Bern University of Applied Sciences, Murtenstrasse 10, 3008 Bern, Switzerland; 2 CTU Bern, and Institute of Social and Preventive Medicine (ISPM), University of Bern, Mittelstrasse 43, CH-3012 Bern, Switzerland; 3 Department of Obstetrics and Gynecology. Inselspital, University of Bern, Bern, Switzerland

**Keywords:** Metabolic disorders, Endocrine reproductive disorders, Epidemiology, Weight management, Risk factors

## Abstract

Maternal obesity often occurs together with comorbid diabetes and hypertensive disorders. All three conditions are independently associated with negative perinatal outcomes. Our objective was to determine the risk and burden of adverse perinatal outcome that could be attributed to maternal obesity in combination with a comorbid status. We analyzed data from 324′664 singleton deliveries in Switzerland between 2005 and 2016. For the association of maternal obesity in the presence or absence of comorbidities with various perinatal outcomes, we estimated adjusted relative risk (RR) using multivariable regression modeling and determined the multivariable-adjusted attributable fraction of the population (AFp). Obesity was a main predictor for macrosomia, fracture of the clavicle, failure to progress in labor and prolonged labor. By stratifying women based on comorbidities, we identified significantly increased risk for preterm birth and early neonatal death only for women diagnosed with a comorbidity. However, various other outcomes were independently associated with either obesity or comorbidities. The AFp showed greatest reduction in comorbidities (15.4/15.0/13.2%), in macrosomia (6.3%) and in shoulder dystocia (4.8%) if all women were to become non-obese. We suggest that comorbidities such as diabetes and hypertensive disorders should be considered when relating maternal obesity to adverse perinatal outcomes.

## Introduction

Obesity is one of the greatest health problems globally and is considered a major cause of death and disease in industrial countries^1^. The epidemic rise of obesity is also reflected in an increased prevalence of maternal obesity. In the US, more than 50% of pregnant women are considered obese, while in Europe, the number of women considered overweight and obese during pregnancy has also increased significantly, attaining rates of 30–37% in 2010^2,3^. One third of these women were considered obese^4^. According to a study by Frischknecht *at al*.^5^, the prevalence of women with obese pre-pregnancy BMI in Switzerland almost doubled between 1986 and 2004.

Obesity, defined as a BMI ≥ 30 kg/m^2^, has been shown to impact mothers’ and infants’ health by increasing the risk of adverse perinatal outcomes such as stillbirth^6^, congenital malformations^7^ and delivery complications^8^. The rise in obesity prevalence is considered to be the major determinant of the striking increase in obesity attendant comorbidities in pregnancy, mainly gestational diabetes and hypertensive disorders^9^. The term comorbidity refers to a situation in which one or more disorders co-occur in the same individual, either at the same time or in some causal sequence^10^. Many women have diabetes or hypertension or both, in addition to being obese. This indicates the existence of a metabolic syndrome. In pregnancy, obesity in combination with comorbidities like pre-existing diabetes, gestational diabetes mellitus (GDM), pre-gestational hypertension, gestational hypertension as well as pre-eclampsia can be present as a mixture of symptoms, modulated by these diverse comorbid conditions and thus complicate a precise diagnosis. Furthermore, the interaction between illnesses can worsen the course of both the obese and the comorbid condition or adversely influence the course of the other condition.

Obesity, diabetes and hypertension have all been discussed independently regarding poor perinatal outcomes^11–13^. Each of these conditions can contribute to adverse perinatal outcomes and might worsen along with the normal physiological changes in pregnancy. As with maternal obesity, pregnant women with pre-existing diabetes or GDM are at increased risk of pregnancy loss, perinatal mortality, fetal macrosomia and congenital malformations^14^. A review of 55 general US population studies demonstrated that hypertensive disorders during pregnancy are also a risk factor for cesarean delivery (41.4%), preterm birth (28.1%), neonatal unit admission (20.5%), and perinatal death (4%)^15^. The complex relationship and the interplay between obesity and its attendant comorbidities and the relative contributions of each to the increased risk of obstetric perinatal complications are still unclear. Despite strong claims for significant association of BMI and various adverse perinatal outcomes, some inconsistency between studies remains^16–18^. The recent umbrella review of 156 meta-analyses provided strong evidence for an association between obesity and only three obstetric outcomes: cesarean delivery (RR 2.00, 95% CI 1.87–2.15), preeclampsia (RR 4.14, 95% CI 3.16–4.75) and low Apgar score (RR 1.29, 95% CI 1.23–1.36)^19^, while other outcomes like fetal death, macrosomia, preterm birth were not clearly linked to obesity. Such inconsistencies may be attributable to the effects of comorbidities on the relative risk for adverse perinatal outcomes, in addition to obesity as a risk factor alone.

The objective of the current study was to investigate the interplay between obesity and comorbidities, diabetes and hypertensive disorders and how these conditions contribute to poor perinatal outcomes. We estimated the adjusted relative risk (RR) for obese women based on the presence or absence of comorbidities and thus quantified an attributable fraction of the population (AFp)^20^. While RR offers an estimate of the strength of an association, AFp considers both the RR and the prevalence of the exposure in the population. It provides information on the population disease load that is due to the underlying exposure of maternal obesity, diabetes and hypertensive disorders. It is essential to estimate the proportion of disease burden that could be prevented by reducing maternal obesity and comorbidities^21^.

## Methods

### Study design and data collection

The present study retrospectively analyzed anonymized data from women in Switzerland who delivered singleton infants between 22 and 43 weeks gestation from January 1, 2005 to December 31, 2016.

The study utilized a database containing details of deliveries collected prospectively by a Swiss obstetric study group (Arbeitsgemeinschaft Schweizerischer Frauenkliniken, Amlikon, Switzerland) during a 12-year period (January 2005 – December 2016)^22^. The group collates and manages data from more than 100 obstetric hospitals of various sizes and structures. The quality of the data recorded was ensured by a two-steps control system. Firstly, the completeness and exactness of all data were verified at each participating center at the time of discharge by a senior obstetrician. Secondly, the plausibility of all data entered in the database was assessed by the data center quality control group. In case of data discrepancy, the hospitals were asked to verify and correct the information, if necessary. All variables included in the database, with the exception of maternal age and weight as well as birth weight were collected as categorical variables (e.g., second stage of labor longer than two hours, maternal hemorrhage greater than 1000 mL). Items in the database contain the International Statistical Classification of Diseases and Related Health Problems, 10^th^ Revision (ICD-10) codes.

Because data were anonymized and irreversibly de-identified, this study did not need approval from the Swiss ethics committee, according to the Swiss Federal Act on Research involving Human Being (810.30, Art. 2, 2)^23^.

### Exposures

Pre-pregnancy weight status was based on the body mass index (BMI), calculated as weight (kg) divided by height (m) squared and registered at the first prenatal visit by the physicians in charge. The physicians further categorized pre-pregnancy BMI by obesity status, defined as non-obese (BMI < 30 kg/m^2^) or obese (BMI ≥ 30 kg/m^2^) according to the World Health Organization’s definition^24^.

Maternal comorbidities according to hospitalization diagnoses were extracted from the database using the following (ICD-10) codes (in brackets): pre-existing diabetes (treated) (E14.9) and gestational diabetes mellitus NOS (O24.4), whereby mothers were only counted in one of the diabetes categories. Hypertensive disorders included gestational hypertension without significant proteinuria ( ≥140/90) (O13), pre-eclampsia (O14, O14.1), eclampsia (O15.9), pre-existing hypertension (O10.9, O11).

### Perinatal outcomes

Labor outcomes included instrumental vaginal delivery methods (vacuum extraction and forceps) (O81), cesarean delivery (primary, secondary and elective section) (O82), induction of labor (physical, systemic/vaginal prostaglandin use), prolonged labor (prolonged first stage >12 h, prolonged second stage >2 h) (O63.0, O63.1), failure to progress in labor >2 h (O63.9), obstructed labor due to shoulder dystocia (O66.0), fetal heart rate anomaly (O68.0), epidural anesthesia. Neonatal outcomes included macrosomia defined as a birth weight of 4000 g or above. 5 minutes Apgar scores ≤7 were defined as abnormal^25,26^. Other neonatal outcomes were transitory neonatal hypoglycemia (P70.4), respiratory distress of newborn (P22.9), fracture of the clavicle (P13.4). Preterm birth was defined as delivery <37 weeks of gestation. Intrauterine fetal death (O36.4), stillbirth and infant death up to seven days post-partum (P95) were defined as early neonatal death^27^.

### Statistical analysis

Prevalence of obesity, diabetes and hypertensive disorders in pregnant women across the time span of 2005–2016 was calculated. All analysis were performed using R version 3.4.1. Logistic regression models provided adjusted relative risks (RR) and 95% confidence intervals for the association of maternal obesity in combination with comorbidities and various complications during pregnancy and delivery. Multivariable Poisson regression models with robust standard errors were used for outcomes with a prevalence ≥10% ^28^. All models were adjusted for maternal age, parity, history of smoking during pregnancy, and ethnicity. Instrumental vaginal delivery was additionally adjusted for cesarean section.

Outcome variables were compared to those of non-obese women or non-obese women without comorbidities as the reference group. Regression models were implemented using generalized linear models (GLM) from the statistical package available in R^29^.

The attributable fraction of the population (AFp) was estimated for the effect of obesity and/or comorbidities on all obstetric outcomes using the prevalence of disease in the total population and the prevalence of disease in those with the risk factor. For each outcome the following standard formula was applied^20^:

$${\rm{AFp}}={\rm{prevalence}}\,{\rm{of}}\,{\rm{risk}}\,{\rm{factor}}\,\ast \,({\rm{RR}}-1)/(\mathrm{prevalence}\,{\rm{of}}\,{\rm{risk}}\,{\rm{factor}}\,\ast \,({\rm{RR}}-{\rm{1}})+1)$$

Nonparametric bootstrap confidence intervals from the boot package available in R were employed to model AFp while incorporating appropriate levels of uncertainty to account for variance in the estimates^30^. A thousand replicate samples were created from the original sample using with-replacement sampling. The replicate samples are used to create a 95% confidence interval (CI) defined as the 2.5^th^ and 97.5^th^ percentile of the distribution of the 1000 possible values^31^.

## Results

A total of 324,664 singleton births over 22 weeks of gestation were recorded in the ASF Database between 2005 and 2016. Complete data, both sociodemographic and clinical, including labor, in particular neonatal outcomes, were available for 324,664 women (99.9%). The clinical characteristics of the study population are depicted in Table 1. A total of 23,456 (7.2%) women were classified as obese with a BMI ≥ 30 kg/m^2^. This sample of 63.1% Swiss, 28.2% European and 8.7% non-European matched the original statistics of a representative sample of adult females in Switzerland (Table 1)^32^.

**Table 1:** Maternal characteristics and perinatal (labor and neonatal) outcomes (n = 324,664).

Maternal characteristics	
Age (years)	31.06 ± 5.08
**Parity**	
1	156,684 (48.3)
2	116,392 (35.8)
3+	51,568 (15.9)
**Origin**	
Swiss	204,903 (63.1)
European	91,581 (28.2)
Non-European	28,180 (8.7)
Smoking during pregnancy	19,759 (6.1)
Pre-pregnancy BMI ≥ 30 kg/m^2^	23,456 (7.2)
- with comorbidities	5,086 (1.6)
Pre-pregnancy BMI < 30 kg/m^2^	301,208 (92.8)
- with comorbidities	19,978 (6.2)
**Labor outcomes**	
Cesarean section	93,869 (28.9)
Epidural anesthesia	89,883 (27.7)
Failure to progress in labor	20,671 (6.4)
Fetal heart rate anomaly	75,478 (23.6)
Induction of labor	62,661 (19.3)
Instrumental vaginal delivery	37,553 (11.6)
Prolonged labor	31,825 (9.8)
Shoulder dystocia	2,202 (0.7)
**Neonatal outcomes**	
5′ Apgar score ≤ 7	17,736 (5.5)
Early neonatal death	1,366 (0.4)
Fracture of the clavicle	563 (0.2)
Intensive care unit admission	14,056 (4.3)
Macrosomia	27,281 (8.4)
Neonatal hypoglycemia (<2 mmol/L)	2,471 (0.8)
Preterm birth (<37 weeks of gestation)	18,411 (5.7)
Respiratory distress of newborn	13,584 (4.2)

Data are mean ± SD or count (%).

Obese women had a higher probability of having at least one comorbidity compared to non-obese women (21.7% in obese vs. 6.6% in non-obese) (Table 2). Their pregnancy was four times as likely to be complicated by hypertensive disorders (8.4% vs. 2.2%). Obese women had GDM diagnosed three times more often (14.1% vs. 4.4%) than their non-obese peers (Table 2).

**Table 2:** Rates of comorbidities in count (%).

	Total	Non-obese	Obese
	(n = 324,664)	(n = 301,208)	(n = 23,456)
=1 comorbidity	22,894 (7.1)	18,488 (6.1)	4,406 (18.8)
>1 comorbidities	2,170 (0.7)	1,490 (0.5)	680 (2.9)
Hypertensive disorders	8,437 (2.6)	6,465 (2.2)	1,972 (8.4)
Pre-existing diabetes	2,415 (0.7)	1,869 (0.6)	546 (2.3)
Gestational diabetes mellitus (GDM)	16,495 (5.1)	13,185 (4.4)	3,310 (14.1)

Next, we analyzed the effect of obesity and obesity stratified based on the presence or absence of comorbidities on perinatal (labor and neonatal) outcomes, expressed as RR occurring relative to that of non-obese women without comorbidities (Table 3). Obesity is significantly associated with all adverse pregnancy outcomes that were assessed in this study except for prolonged labor, early neonatal death and preterm birth. The RRs were highest for hypertensive disorders (RR 4.01), pre-existing diabetes (RR 3.83), and GDM (RR 3.24). Furthermore, RRs of macrosomia in obese women almost double regardless of comorbidities diagnosed. A similar pattern of significant association for women with obesity was observed for fracture of the clavicle, failure to progress in labor, prolonged labor, instrumental vaginal delivery and epidural anesthesia. The RRs were lower for these outcomes when obese women were additionally affected by a comorbidity.

**Table 3:** Adjusted relative risks (RR) with 95% confidence intervals (CI) for the association between obesity, comorbidities and adverse labor and neonatal outcomes in singleton deliveries of women between 2005 and 2016 (n = 324,664).

	aRR^a^ (95% CI)			
	total obese	non-obese comorbid	obese non-comorbid	obese comorbid
**Comorbidities**				
Hypertensive disorders	**4**.**01** (3.81,4.22)			
Pre-existing diabetes	**3**.**83** (3.48,4.20)			
Gestational diabetes mellitus (GDM)	**3**.**24** (3.11,3.36)			
**Labor outcomes**				
Cesarean section	**1**.**55** (1.52,1.58)	**1**.**41** (1.37,1.44)	**1**.**52** (1.48,1.56)	**1**.**84** (1.76,1.91)
Epidural anesthesia	**1**.**05** (1.02,1.07)	**1**.**01** (0.98,1.04)	**1**.**07** (1.04,1.10)	**0**.**99** (0.93,1.04)
Failure to progress in labor	**1**.**48** (1.42,1.54)	**0**.**94** (0.89,0.99)	**1**.**54** (1.47,1.61)	**1**.**25** (1.13,1.37)
Fetal heart rate anomaly	**1**.**09** (1.06,1.12)	**1**.**06** (1.03,1.09)	**1**.**12** (1.09,1.15)	**1**.**01** (0.95,1.07)
Induction of labor	**1**.**65** (1.60,1.69)	**2**.**06** (2.01,2.11)	**1**.**54** (1.49,1.59)	**2**.**60** (2.49,2.71)
Instrumental vaginal delivery^‡^	**1**.**06** (1.02,1.11)	**1**.**02** (0.98,1.07)	**1**.**07** (1.02,1.12)	**1**.**02** (0.93,1.13)
Prolonged labor	**1**.**04** (1.00,1.09)	**0**.**86** (0.82,0.91)	**1**.**07** (1.02,1.13)	**0**.**89** (0.81,0.91)
Shoulder dystocia	**1**.**69** (1.46,1.95)	**1**.**74** (1.49,2.01)	**1**.**53** (1.29,1.80)	**2**.**78** (2.14,3.55)
**Neonatal outcomes**				
5′ Apgar score ≤7	**1**.**17** (1.11,1.23)	**1**.**40** (1.33,1.45)	**1**.**08** (1.02,1.15)	**1**.**60** (1.46,1.75)
Early neonatal death	**1**.**08** (0.88,1.31)	**1**.**30** (1.06,1.57)	**0**.**94** (0.73,1.18)	**1**.**68** (1.19,2.29)
Fracture of the clavicle	**1**.**61** (1.20,2.12)	**1**.**06** (0.72,1.51)	**1**.**65** (1.20,2.23)	**1**.**47** (0.70,2.67)
Intensive care unit admission	**1**.**22** (1.16,1.29)	**2**.**03** (1.93,2.13)	**1**.**08** (1.01,1.16)	**2**.**10** (1.92,2.29)
Macrosomia	**1**.**93** (1.86,2.00)	**1**.**11** (1.05,1.16)	**1**.**96** (1.89,2.04)	**1**.**84** (1.70,1.99)
Neonatal hypoglycemia (<2 mmol/L)	**1**.**64** (1.45,1.86)	**4**.**28** (3.88,4.71)	**1**.**26** (1.05,1.49)	**4**.**63** (3.91,5.44)
Preterm birth (<37 weeks of gestation)	**0**.**99** (0.94,1.05)	**2**.**34** (2.26,2.44)	**0**.**81** (0.76,0.87)	**2**.**03** (1.88,2.20)
Respiratory distress of newborn	**1**.**34** (1.27,1.41)	**1**.**79** (1.70,1.89)	**1**.**23** (1.15,1.32)	**2**.**03** (1.85,2.23)

^a^Poisson (prevalence >10%) or logistic (prevalence <10%) models adjusted for age, ethnicity, parity and history of smoking during pregnancy. ^‡^Additionally adjusted for cesarean delivery.

In contrast, RRs for other outcomes including preterm birth, intensive care unit admission, neonatal hypoglycemia (glucose <2 mmol/L), 5′ Apgar score ≤7 and early neonatal death were highest when women suffered from comorbidities. Non-obese women with comorbidities showed a similar significant pattern of risk for these adverse perinatal outcomes as their obese peers. This suggests an association with a comorbid condition, rather than with an obese status.

Further outcomes such as cesarean delivery, induction of labor, shoulder dystocia and respiratory distress of newborn were independently associated with both obesity and comorbidities. Highest RRs were identified for obese women suffering from comorbidities.

The attributable fractions of the population (AFp) showed 15.4% of hypertensive disorders, 15.0% of pre-existing diabetes and 13.2% of GDM may potentially be prevented if all women became non-obese (Table 4). Assuming this scenario, the greatest reductions of perinatal (labor and neonatal) outcomes would be expected for macrosomia (6.3%), shoulder dystocia (4.8%), induction of labor (4.5%), neonatal hypoglycemia (4.5%) and fracture of the clavicle (4.3%). At the same time, however, for neonatal hypoglycemia, intensive care unit admission, and preterm birth, the potential benefit would be greater if morbidities (diabetes and hypertensive disorders) rather than obesity alone were eliminated. If obese women without comorbidities were to become non-obese, macrosomia may be potentially reduced to 5.2%, similar to the effect of eliminating obesity of obese women with comorbidities for neonatal hypoglycemia.

**Table 4:** Attributable Fraction in the population (AFp) with 95% confidence intervals (CI) for the risk factors with a variable combining obesity and comorbidities.

	AFp^a^ [%] (95% CI)			
	total obese	non-obese comorbid	obese non-comorbid	obese comorbid
**Comorbidities**				
Hypertensive disorders	**15**.**4** (14.5,16.4)			
Pre-existing diabetes	**15**.**0** (13.3,16.7)			
Gestational diabetes mellitus (GDM)	**13**.**2** (12.6,13.8)			
**Labor outcomes**				
Cesarean section	**3**.**8** (3.6,3.9)	**2**.**4** (2.3,2.6)	**2**.**9** (2.7,3.0)	**1**.**3** (1.2,1.4)
Epidural anesthesia	**0**.**6** (0.5,0.8)	**0**.**2** (0.1,0.4)	**0**.**6** (0.4,0.7)	**0**.**1** (0.0,0.1)
Failure to progress in labor	**1**.**1** (0.8,1.4)	**−1**.**5** (−1.7,−1.2)	**1**.**2** (0.9,1.5)	**−0**.**2** (−0.3,−0.1)
Fetal heart rate anomaly	**0**.**7** (0.6,0.9)	**0**.**4** (0.3,0.6)	**0**.**7** (0.6,0.9)	**0**.**0** (0.0,0.1)
Induction of labor	**4**.**5** (4.2,4.7)	**6**.**1** (5.9,6.4)	**3**.**0** (2.8,3.2)	**2**.**4** (2.3,2.6)
Instrumental vaginal delivery^‡^	**0**.**4** (0.1,0.7)	**0**.**1** (−0.1,0.4)	**0**.**4** (0.1,0.6)	**0**.**0** (0.0,0.2)
Prolonged labor	**0**.**8** (0.5,1.2)	**−0**.**5** (−0.8,−0.3)	**0**.**8** (0.5,1.1)	**0**.**0** (−0.2,0.1)
Shoulder dystocia	**4**.**8** (3.2,6.2)	**4**.**3** (2.9,5.8)	**2**.**9** (1.6,4.3)	**2**.**7** (1.7,3.8)
**Neonatal outcomes**				
5′ Apgar score ≤7	**1**.**2** (0.7,1.6)	**2**.**4** (2.0,2.8)	**0**.**5** (0.1,0.8)	**0**.**9** (0.7,1.2)
Early neonatal death	**1**.**0** (−0.2,2.4)	**1**.**8** (0.2,3.3)	**−0**.**4** (−1.7,0.9)	**1**.**1** (0.2,1.9)
Fracture of the clavicle	**4**.**3** (1.8,7.4)	**0**.**4** (−2.1,2.8)	**3**.**6** (0.8,6.3)	**0**.**7** (−0.8,2.2)
Intensive care unit admission	**1**.**6** (1.2,2.0)	**5**.**9** (5.4,6.5)	**0**.**5** (0.0,0.9)	**1**.**7** (1.4,2.0)
Macrosomia	**6**.**3** (5.9,6.7)	**0**.**6** (0.3,1.0)	**5**.**2** (4.8,5.5)	**1**.**3** (1.1,1.5)
Neonatal hypoglycemia (<2 mmol/L)	**4**.**5** (3.1,5.7)	**16**.**8** (15.0,18.6)	**1**.**4** (0.2,2.6)	**5**.**4** (4.3,6.5)
Preterm birth (<37 weeks of gestation)	**0**.**0** (−0.4,0.3)	**7**.**7** (7.2,8.2)	**−1**.**1** (−1.4,−0.7)	**1**.**6** (1.3,1.8)
Respiratory distress of newborn	**2**.**4** (1.9,2.8)	**4**.**6** (4.2,5.2)	**1**.**3** (0.8,1.7)	**1**.**6** (1.3,1.9)

^a^Attributable fraction in the population adjusted for age, ethnicity, parity and history of smoking during pregnancy. ^‡^Additionally adjusted for cesarean delivery.

## Discussion

Using population-based data of women and their offspring from Switzerland, we examined the impact of obesity and its attendant comorbidities including diabetes and hypertensive disorders on perinatal outcomes. Our results confirm that a link exists between obesity and the risk of adverse perinatal outcomes. This concurs with the findings of a recent meta-analysis, which identified maternal BMI over 30 as a strong, or at least highly suggestive risk factor for fetal macrosomia, low Apgar score, instrumental vaginal delivery, gestational diabetes mellitus and pre-eclampsia^19^.

Maternal obesity, diabetes and hypertensive disorders seem to be closely linked and often occur in the same patient. They also independently increase the health risk for mother and fetus/infant^33^. Despite the indications of interaction, little is known about the relative risk contribution of maternal obesity, together with comorbid diabetes and hypertensive disorders to adverse perinatal outcomes. We showed that macrosomia, fracture of the clavicle, failure to progress in labor and prolonged labor are significantly associated with maternal obesity but were only slightly influenced by comorbidities. Similarly, a study found maternal hyperglycemia and obesity to be independent predictors for various adverse perinatal outcomes^34^. By demonstrating distinct higher risks for overweight women, with or without GDM, they confirm the relevance of increased weight independently of hyperglycemia. This might be explained by the fact that diabetes and hypertensive disorders are manageable components during pregnancy.

Not only does glycemic control in diabetes or antihypertensive treatment reduce the impact of these comorbidities, but they may even be beneficial for the biological mechanisms of parturition in obese women^35^. Indeed, we observed that risks for epidural anesthesia, failure to progress in labor and prolonged labor are slightly but significantly lower in obese women suffering from a comorbidity, compared to obesity alone. The observed protective benefit might be due to an unidentified effect of the comorbidity treatment or, to a subtle change in compliance with a diet that accompanied a specific therapy. Obese women with GDM who achieved desired levels of glycemic control using insulin therapy had similar macrosomia rates to normal-weight controls. However, this “positive” effect was eliminated by exclusively diet-controlled therapy^36^.

In contrast, for some other perinatal adverse outcomes we identified increased risks only when women presented additional comorbidities. For example, we showed that obese women with comorbidities exhibit more than two-fold risk for preterm birth. However, preterm birth was apparently less prevalent when obese women did not suffer from any comorbidity. Tsur *et al*.^37^ showed a comparable protective effect of obesity on the risk of preterm birth. Stratification of obese women with and without comorbidities resulted in a decreased risk of preterm birth associated with obesity, independent of comorbidities. The authors argued that fat tissue confers protection for preterm birth through alteration of metabolic factors such as tumor necrosis factor alpha or obesity-associated gene FTO variants^37^. However, a Spanish study linking overweight to GDM confirmed the positive correlation of glucose intolerance and preterm deliveries, independent of the mother’s BMI, but it did not show a preventive role of obesity^34^. A possible explanation is the adjustment of preterm birth prevalence for macrosomia, which is known to be a factor linked to post-maturity^38^. Likewise, in our study, diabetes and hypertensive disorders rather than obesity appear to be exclusive risk factors for intensive care unit admission and early neonatal death. Even though recent reviews identified an elevated risk for intensive care unit admission (OR 1.5) and neonatal death (RR 1.46) associated with obesity, they agreed that maternal obesity itself, increases the risk for comorbidities that are risk factors for stillbirth, preterm birth and subsequently intensive care unit admission^39,40^.

Here, both obesity and comorbidities can be considered as independent risk factors for outcomes including cesarean section, induction of labor, shoulder dystocia, neonatal hypoglycemia, and respiratory distress of newborn. For example, it has been shown in multiple studies that the excessive risk for cesarean section among women is irrespective of whether they are either obese, have diabetes, hypertension, or a combination of these conditions^8,41,42^. Indeed, totally unrelated events can also require cesarean section. On the one hand, the cephalo-pelvic disproportion of macrosomic babies of obese mothers can lead to non-progressive labor, resulting in emergency cesarean section^43^. On the other hand, increased blood pressure in the mother and preeclampsia, often results in intra-uterine growth restriction, and increases the cesarean section rates to over 50%^44,45^.

RR signifies the association between an exposure and an outcome, while taking the incidence in the exposed group into consideration. AFp constitutes the difference in proportion of incidence between exposed and unexposed groups. It can therefore be considered as the preventive potential on an outcome when exposure is reduced. We took advantage of our large and comprehensive dataset to determine how the burden of obesity in combination with its comorbidities could be attributed to adverse perinatal outcomes.

Generally, maternal obesity may potentially be the triggering factor of 4.5% of neonatal hypoglycemia (Table 4). However, obesity alone contributed only to 1.4% of neonatal hypoglycemia, whereas, when combined with comorbidities, increased to 5.4% (Table 4). The contribution to neonatal hypoglycemia of comorbidities in obesity seems striking when we consider that in this study population, the prevalence of obese women without comorbidities was almost four times greater when compared to their peers who suffered from comorbidities (5.6% and 1.6%). This highlights why it is particularly important to consider the comorbidities of diabetes and hypertensive disorders when looking at the health burden of maternal obesity.

The population studied here exhibits a comparatively low maternal obesity prevalence (7.2%) compared to most of other studies published (>20%)^1,46–48^. Therefore, only a few perinatal outcomes may be attributable to maternal obesity of more than 4%. Nevertheless, reducing maternal obesity will not only be beneficial for preventing a subset of adverse outcomes, but positively impact all perinatal outcomes by lowering the prevalence of comorbidities. Our data show that a hypothetical elimination of maternal obesity would lead to an approximate 15% reduction in each comorbidity. With increased obesity prevalence there is even greater potential in decreasing comorbidities through obesity prevention. In Canada, where obesity prevalence is 25%, half of the GDM and almost a third of hypertensive disorders in pregnancy could be avoided by eliminating maternal obesity^48^. Consequently, up to 50% of adverse perinatal outcomes associated with comorbidities could be prevented, if maternal obesity was eliminated there.

Our study took advantage of a unique standardized dataset from a large, consistently collected, representative sample of women provided by Swiss hospitals. However, stratifying women with comorbidities might create a selection bias known as collider stratification bias^37,49^. In this case, diabetes and hypertensive disorders may be potential colliders affected both by obesity and factors, which were not controlled for. RR of women without comorbidities may be affected by these factors, due to a possible uneven selection of women without these factors^50^. Such factors could include socio-economic parameters such as household income, employment status and level of education not accessible to control for in this study^51^. More work is necessary to explore the exact extent of additional cofounders’ influences, which was not considered here. Nevertheless, we addressed major confounding factors such as age, parity, ethnicity and history of smoking.

## Conclusions

Using a large unique and representative dataset of women giving birth in Switzerland from 2005 to 2016, we showed that maternal obesity is strongly associated with comorbid diabetes and hypertensive disorders. These comorbidities further impact on a variety of adverse perinatal outcomes not necessarily directly linked to obese conditions. Obesity attendant comorbidities can act independently and should be considered when associating obesity with dysfunctional labor. Therefore, interventional study designs related to obesity in pregnancy should attempt to distinguish the effect of various comorbidities.

From a public health perspective, the AFp of maternal obesity in the current study can help illustrate that the burden of obesity in a comparatively low-prevalence population support potential adverse outcomes among the women affected. Efforts to prevent adverse perinatal outcomes by reducing obesity may need to focus on these women in order to be most effective.

## Data Availability

The datasets analyzed during the current study are available from the corresponding author on reasonable request.
